# Baseline Residual Kidney Function and Its Ensuing Rate of Decline Interact to Predict Mortality of Peritoneal Dialysis Patients

**DOI:** 10.1371/journal.pone.0158696

**Published:** 2016-07-08

**Authors:** Miguel Pérez Fontán, César Remón Rodríguez, Marta da Cunha Naveira, Mercè Borràs Sans, Carmen Rodríguez Suárez, Pedro Quirós Ganga, Emilio Sánchez Alvarez, Ana Rodríguez-Carmona

**Affiliations:** 1 Division of Nephrology, University Hospital of A Coruña, A Coruña, Spain; 2 Division of Nephrology, University Hospital Puerta Real, Cádiz, Spain; 3 Division of Nephrology, University Hospital Arnau de Vilanova, Lleida, Spain; 4 Division of Nephrology, University Hospital of Asturias, Oviedo, Spain; The University of Tokyo, JAPAN

## Abstract

**Background:**

Baseline residual kidney function (RKF) and its rate of decline during follow-up are purported to be reliable outcome predictors of patients undergoing Peritoneal Dialysis (PD). The independent contribution of each of these factors has not been elucidated.

**Method:**

We report a multicenter, longitudinal study of 493 patients incident on PD and satisfying two conditions: a glomerular filtration rate (GFR) ≥1 mL/minute and a daily diuresis ≥300 mL. The main variables were the GFR (mean of urea and creatinine clearances) at PD inception and the GFR rate of decline during follow-up. The main outcome variable was patient mortality. The secondary outcome variables were: PD technique failure and risk of peritoneal infection. The statistical analysis was based on a multivariate approach, placing an emphasis on the interactions between the two main study variables.

**Main Results:**

Baseline GFR and its rate of decline performed well as independent predictors of both patient mortality and risk of peritoneal infection. These two main study variables maintained a moderate correlation with each other (r^2^ = 0.12, p<0.0005), and interacted clearly, as predictors of patient mortality. A low baseline GFR followed by a fast decline portended the worst survival outcome (adjusted HR 3.84, 95%CI 1.81–8.14, p<0.0005)(Ref. baseline GFR above median plus rate of decline below median). In general, the rate of decline of RKF had a greater effect on mortality than baseline GFR, which had no detectable effect on survival when the decline of RKF was slow (HR 1.17, 95% CI 0.81–2.22, p = 0.22). Conversely, a relatively high GFR at the start of PD still carried a significant risk of mortality, when RKF declined rapidly (HR 1.89, 95% CI 1.05–3.72, p = 0.028).

**Conclusion:**

The risk-benefit balance of an early versus late start of PD cannot be evaluated without taking into consideration the rate of decline of RKF. This circumstance may contribute to explain the controversial results observed at the time of evaluating the potential benefits of an early initiation of PD.

## Introduction

Preservation of residual kidney function (RKF) facilitates management and improves quality of life of patients treated with chronic peritoneal dialysis (PD). The residual function permits an incremental dosing of PD, which is generally easier to reconcile with the lifestyle of the patients [1], and improves toxin removal and volume control, compared to PD alone [2]. Many observational studies have shown that sustained RKF improves both patient and PD technique survival [3], and has a beneficial effect on other complications of chronic kidney disease and PD therapy, such as cardiovascular disease, disorders of bone and mineral metabolism, inflammation and malnutrition [2].

Previous studies investigating the prognostic value of RKF in PD patients have defined this study variable in different ways, including baseline [4–8] and mean [4,7,9] GFR levels, GFR values antedating selected outcomes [8] or time-dependent strategies [10–13]. Some of these studies have restricted their analyses to patients starting PD, while others have also recruited established PD patients. There is a remarkable paucity of studies comparing the prognostic importance of baseline RKF and its time course during follow-up. It is clear that these variables may influence independently the outcome of PD patients, but it is also conceivable that they have interactive outcome effects. If so, the interaction could help to explain the variable effect of early initiation on the outcome of PD [14,15].

To test this hypothesis, we have undertaken a multicenter, observational study, comparing the effects of baseline RKF and its rate of decline during follow-up on the outcome of patients undergoing PD therapy.

## Population and Method

### General design

We analyzed a historic cohort of patients placed on PD therapy in four Spanish units during the period extending since January 2000 to December 2010, using a multicenter, observational design. The main objective of the study was to establish the prognostic value of both the baseline levels and the rate of decline of RKF during follow-up (main study variables). The main outcome variable was patient mortality. Secondary outcome variables included PD technique failure (defined by drop-out to hemodialysis therapy for at least 90 days) and the rate of peritoneal infection (survival to first episode). We took into consideration the main control variables with a claimed effect on the selected outcomes (see below).

The study complied with the ethical conditions stipulated by the participating centers for retrospective observational studies. The Institutional Investigation Committees of the University Hospital Puerta Real (Cadiz) and of the regional Government of Asturias (Spain) evaluated and approved the study protocol. Approval by these committees requested oral informed consent from the participants who were accessible at the time the study was initiated. This consent was registered in the clinical records of the patients.

### Study population

We included in our analysis all the consecutive cases from the clinical records of the participating units that fulfilled the following inclusion/exclusion criteria:

1. Age older than 10 years at the inception of PD
2. Chronic kidney disease stage 5d
3. Patients incident on PD, either primarily or following hemodialysis therapy or a failed kidney transplant
4. Minimum follow-up of 6 months on PD
5. Estimations of RKF available at least at baseline and after 6 months on PD
6. Baseline 24-hour urine volume ≥300 mL/24 hours and estimated glomerular filtration rate (GFR) ≥1 mL/minute
7. Full clinical records available

### Study variables and data analysis

As previously stated, study variables were baseline RKF and its rate of decline after PD was started. We estimated RKF from the mean of urea and creatinine renal clearances (GFR), at inception of PD (meaning less than one month after initiation of dialysis) and then after 6, 12 and 24 months of PD therapy. We estimated the rate of decline of RKF by calculating the difference between its baseline value and that observed at the time of the last available estimation of GFR (6, 12 or 24 months), and then dividing it by the time of follow-up (months).

Control variables included a wide set of demographic, clinical, biochemical, adequacy and prescription factors, shown in Tables 1 and 2. Body mass index was calculated as body weight (Kg) / Height^2^ (m). Charlson’s score was used to categorize general comorbidity at the initiation of PD. Biochemical determinations were produced mostly with the help of autoanalyzers. C reactive protein levels were estimated by immunoturbidimetry.

**Table 1:** Clinical and demographic characteristics of the study population.

Age (years)	58.1±15.8
Gender (males/females)(%)	314/179 (63.7/36.3)
Kidney disease (%)	
Glomerular	74 (15.0)
Interstitial	51 (10.3)
Vascular	52 (10.5)
Cystic	31 (6.3)
Systemic	6 (1.2)
Diabetic nephropathy	144 (29.2)
Other/Unknown	135 (27.4)
Diabetes mellitus (%)	189 (38.3)
Body mass index (Kg/m^2^)	26.8 ± 4.8
Origin (%)	
Incident on renal replacement therapy	454 (92.1)
Hemodialysis	22 (4.5)
Failed kidney transplant	17 (3.4)
Charlson’s comorbidity score	4.5 ± 2.2
Systolic blood pressure (mm Hg)	136.0 ± 20.4
Diastolic blood pressure (mm Hg)	77.0 ± 12.7
Hemoglobin (g/dL)	11.3 ± 1.6
Plasma albumin (g/L)	36.5 ± 5.3
Plasma cholesterol (mg/dL)	174.4 ± 50.3
C reactive protein (mg/dL)	0.50 (0.24/1.20)
GFR (mL/minute)	7.5 ± 3.3
Diuresis (mL/24 hours)	1437 ± 665
Proteinuria (g/24 hours)	1.7 ± 2.4
D/P creatinine at 240’ (baseline PET)	0.66 ± 0.13
Modality of PD at inception (CAPD/APD)(%)	368/125 (74.6/25.4)
Type of PD solution (low GDPs)(%)	187 (38.2)

Continuous variables expressed as mean ± SD, except C reactive protein (median with interquartile range). Categorized variables expressed as number of cases (%). GFR: Glomerular filtration rate (mean of urea and creatinine renal clearances). PET: Peritoneal equilibration test. GDPs: Glucose degradation products.

**Table 2:** Study population. Time-dependent variables.

	Baseline	6 months	12 months	24 months
N	493	493	419	287
Modality of PD (patients on automated PD)(%)	125 (25.2)	**161 (34.3)**	**161 (38.4)**	**120 (42.0)**
Peritoneal glucose load (g/24 hours)	90 ± 39	**97 ± 48**	**102 ± 53**	**114 ± 61**
Icodextrin for long dwell (%)	287 (58.6)	**308 (65.7)**	**283 (67.9)**	**197 (69.9)**
Number of antihypertensives	1.7 ± 1.1	1.7 ± 1.2	1.7 ± 1.2	1.6 ± 1.2
RAS antagonists (%)	222 (45.3)	212 (44.9)	189 (46.0)	125 (44.0)
Dose of furosemide (mg/24 hours)	37.1 ± 44.6	**62.7 ± 60.8**	**72.0 ± 66.6**	**70.4 ± 68.9**
Systolic BP (mm Hg)	136 ± 19	134 ± 19	133 ± 18	132 ± 18
Diastolic BP (mm Hg)	77 ± 12	76 ± 11	75 ± 11	74 ± 10
D/P creatinine 240’	0.66 ± 0.13	-	0.67 ± 0.12	0.66 ± 0.13
Ultrafiltration (mL/24 h)	888 ± 778	**982 ± 655**	**1040 ±626**	**1118 ± 632**
GFR (mL/minute)	7.5 ± 3.3	**6.8 ± 3.6**	**5.7 ± 3.8**	**4.6 ± 3.9**
Proteinuria (gr/24 h)	1.7 ± 2.4	-	**1.0 ± 1.3**	**0.8 ± 1.1**

Continuous variables denote mean (SD), Categorized variables denote number of cases (%). RAS: Renin-angiotensin system. BP: Blood pressure. D/P: Quotient dialysate/plasma. GFR: Glomerular filtration rate (mean of urea and creatinine renal clearances). Bold characters indicate significant differences versus baseline

Basic univariate data analysis was produced using common parametric (Student’s t test, ANOVA, repeated measures) and nonparametric (χ^2^ distribution, Mann Whitney, Spearman) tests, as appropriate. Univariate survival analyses were generated according to Kaplan Meier plots (log rank test), after categorizing baseline GFR according to median values and its time course by tertiles. Multivariate analysis was based on baseline and time-dependent Cox’s models. Patients were censored in cases of kidney transplant, dialysis withdrawal after improvement of GFR, loss to follow-up, drop-out to hemodialysis (patient survival and peritoneal infection) and demise (technique failure and peritoneal infection).

To explore the effects of baseline and RKF changes, we analyzed their contributions as independent variables in the general Cox’s model. We applied interaction terms to scrutinize potential effect modifications among the main study variables. When statistical significance was reached, we performed additional stratified analyses, to clarify the significance of the findings.

We used the SPSS 19.0 and Stata v.10 softwares for data management.

## Results

### 1. Overview

**Fig 1:**
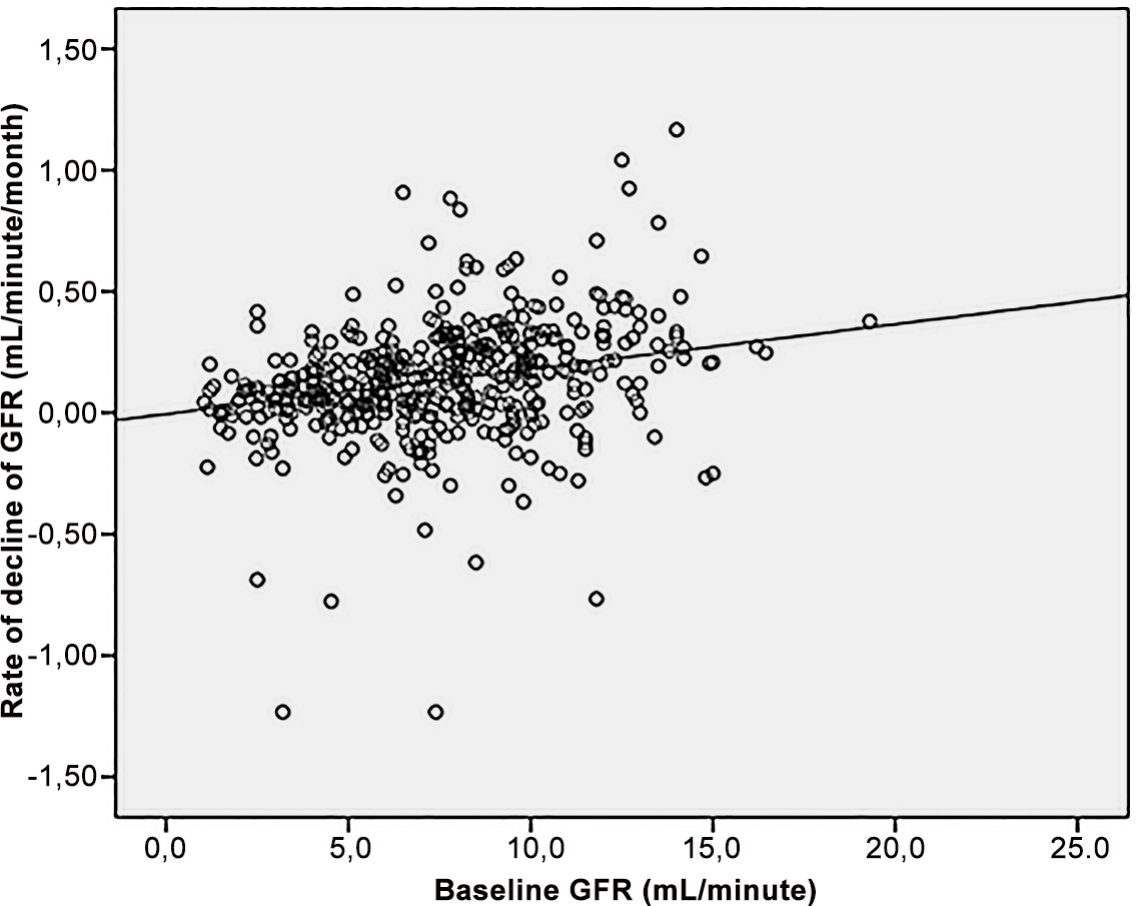
Correlation between baseline GFR and its rate of decline during follow-up on PD.

Six hundred and two patients were pre-selected for the study, but 109 were finally excluded, because they did not fulfill the pre-established criteria for baseline GFR (n = 62) or because of inconsistencies or gaps in their clinical records (n = 40). The study population thus included 493 patients, of whom 287 (58.2%) completed at least 24 months of follow-up (mean 29.9 ± 19.8 months). The main baseline demographic, clinical and time-dependent variables are presented in Tables 1 and 2. Baseline GFR showed a moderate, but significant correlation with its ensuing rate of decline (Fig 1, r^2^ = 0.12, p<0.0005). The mean rate of decline of GFR was 0.13 ± 0.23 mL/minute/month (range -1.23 to 1.17). In 90 cases (18.2%) GFR was higher at the end of follow-up (6–24 months) than at baseline, although only in 24 of them (4.9%) the slope of recovery was faster than 0.2 mL/minute/month.

At the end of follow-up, 149 patients (30.2%) had died. The main causes for death included cardiovascular events (n = 66) and infections (n = 27); 17 patients died for unknown causes. Sixty-nine patients (14.0%) switched to hemodialysis during follow-up; peritoneal infections (n = 27) and inadequacy of PD (n = 16) were the main quoted causes. One hundred and thirty-four patients (27.2%) were censored after undergoing kidney transplantation. One hundred and eighty-eight patients (38.5%) suffered at least one peritoneal infection during follow-up, for a total of 574 episodes (incidence: one episode every 25.7 patient-months).

### 2. Patient mortality

**Fig 2:**
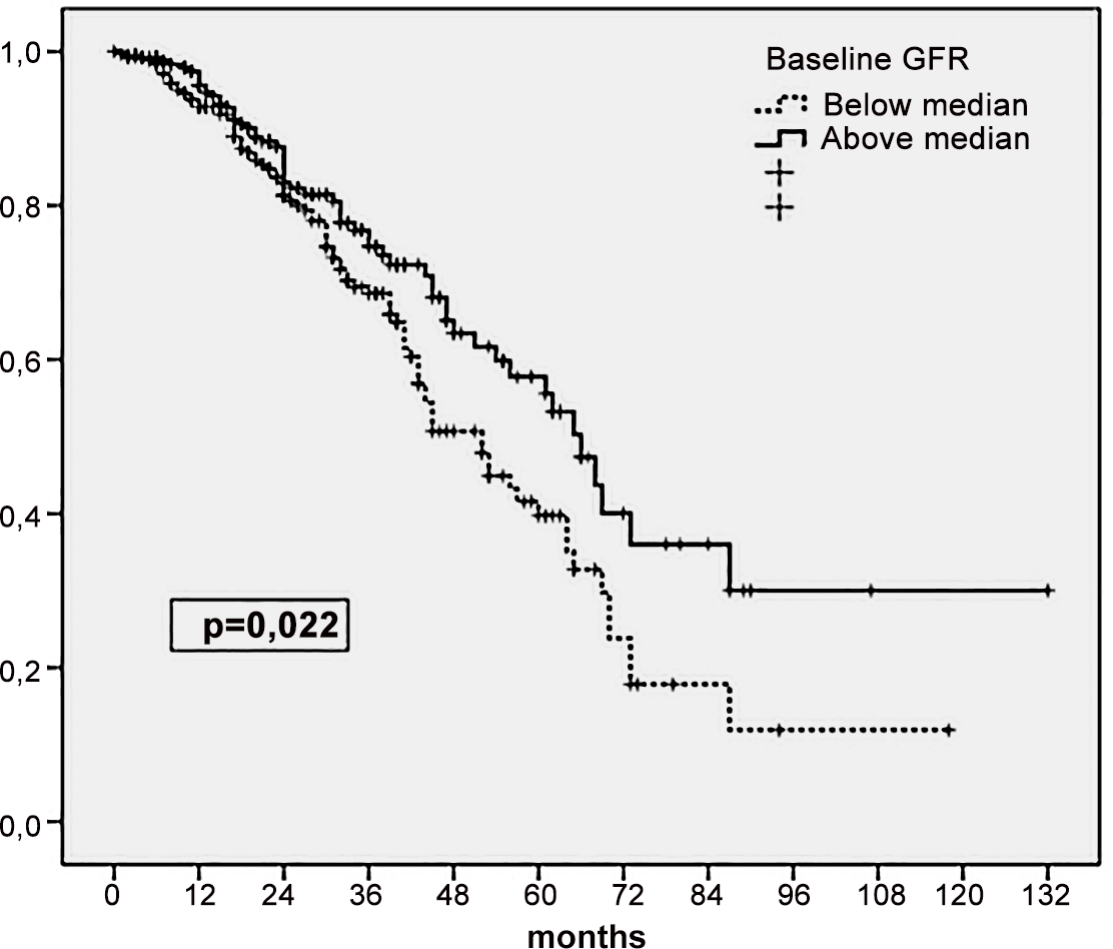
Patient survival according to baseline GFR, categorized from median value. Kaplan Meier plot (log rank).

**Fig 3:**
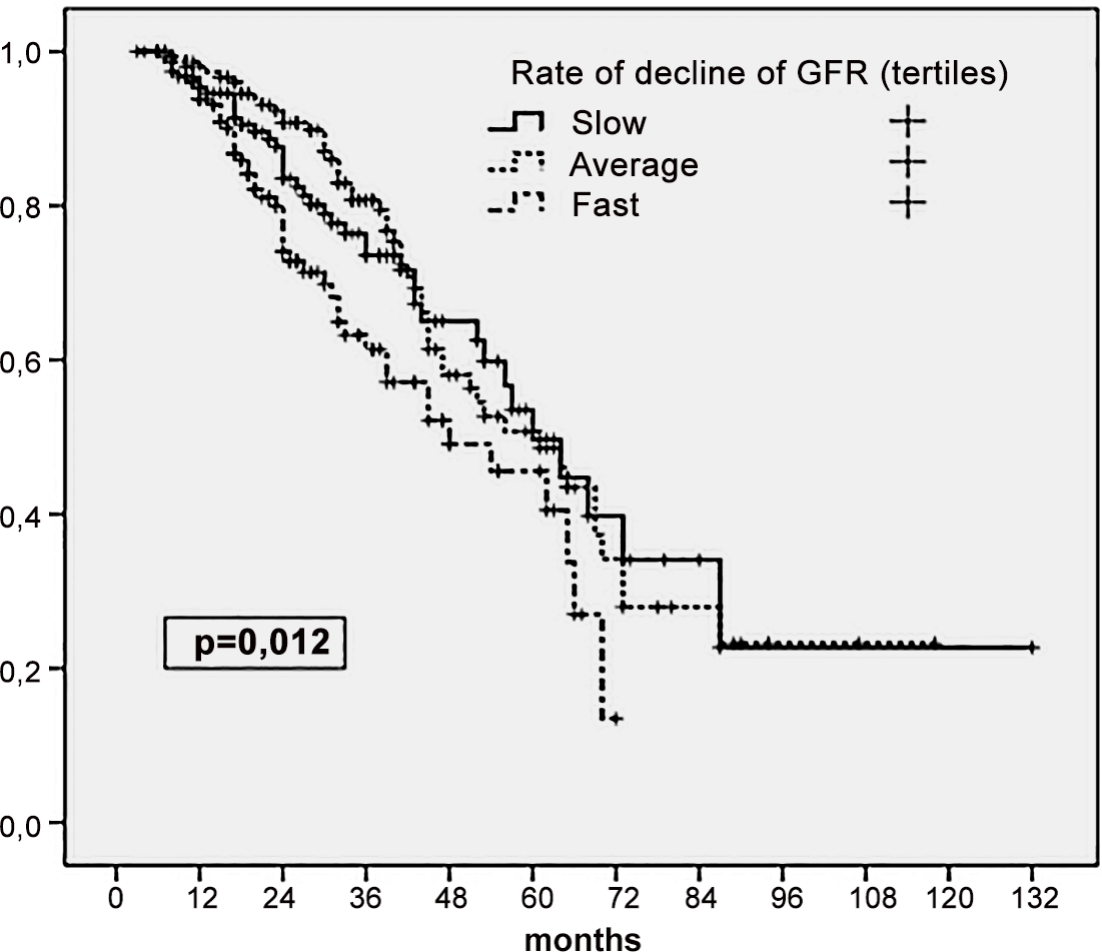
Patient survival according to tertiles of the rate of decline of GFR during follow-up. Kaplan Meier plot (log rank).

Univariate analysis demonstrated that both baseline GFR (Fig 2) and its rate of decline while on PD (Fig 3) correlated with patient mortality. The general Cox’s model confirmed that both variables had an independent effect on mortality (Table 3). Remarkably, we observed a significant interaction between the main study variables, at the time of predicting mortality (p = 0.043 for the interaction term). Subsequent stratified analysis showed that baseline GFR was a predictor of mortality only if its subsequent decline while on PD was relatively rapid. In contrast, the negative prognostic significance of a fast decline of GFR was more apparent if the initial RKF was relatively poor (Table 3). The association of a baseline GFR below median and a pace of decline above median were the worst combination for expected survival (Table 4). The risk of mortality was indeed increased in any setting where GFR decreased rapidly, even if baseline RKF was relatively good. On the contrary, a baseline GFR below median did not portend a poor prognosis, provided that its rate of decline was slow or negative (Table 4). Incidentally, we did notice a significant interaction between age and the rate of decline of GFR (p = 0.032 for the term), suggesting that the impact of the latter variable on mortality was greater in younger (<65 years) (hazard ratio HR 1.08 per year, 95% confidence interval CI 1.04–1.11, p<0.0005) than in older (>65 years) patients (HR 1.04, 95% CI 0.99–1.07, p = 0.068).

**Table 3:** Predictors of mortality of patients starting PD. Multivariate analysis.

	HR	95% CI	P
Age (x year)	1.05	1.03–1.06	<0.0005
Charlson’s score (x point)	1.22	1.10–1.37	<0.0005
Diabetes	1.69	1.15–2.48	0.007
Plasma albumin (x g/L)	0.95	0.92–0.98	0.003
Hemoglobin (x g/dL)	0.88	0.77–1.00	0.053
Peritoneal transport (PET)(x 0,1)	1.02	1.00–1.04	0.007
Rate of decline of GFR (x mL(/min/month)	1.91	1.43–2.56	<0.0005
Baseline GFR above median	1.82	0.92–3.60	0.08
Baseline GFR below median	2.55	1.50–4.34	0.001
Baseline GFR (x mL/min)	0.90	0.84–0.97	0.005
Rate of decline faster than median	0.84	0.75–0.93	0.002
Rate of decline slower than median	0.96	0.88–1.06	0.96

General Cox’s model. Dependent variable: Mortality (any cause). GFR: Glomerular filtration rate (mean of urea and creatinine renal clearances). PET: Peritoneal equilibration test. For baseline GFR and its rate of decline, we present the general coefficients of the model, and then stratified values, to facilitate clinical interpretation of the interaction between both variables.

**Table 4:** Combined effect of baseline GFR and its rate of decline on mortality during follow-up on PD. Stratified analysis.

	n	Unadjusted	Adjusted*
		HR (95% CI) p	HR (95% CI) p
High baseline GFR & Slow decline	97	1 (Ref)	1 (Ref)
High baseline GFR & Fast decline	150	1.78 (0.99–3.37) 0.065	1.89 (1.05–3.72) 0.028
Low baseline GFR & Slow decline	149	1.55 (0.78–3.08) 0.35	1.17 (0.81–2.22) 0.22
Low baseline GFR & Fast decline	97	3.23 (1.66–6.28) 0.001	3.84 (1.81–8.14) <0.0005

Baseline GFR and rate of decline categorized from median values. GFR: Glomerular filtration rate (mean of urea and creatinine renal clearances).

* Control variables: Age, Charlson’s score, diabetes, plasma albumin and peritoneal transport rate

Time-dependent Cox’s model confirmed that GFR, when managed as a time-dependent variable, was an independent predictor of patient mortality (Table 5).

**Table 5:** Predictors of mortality of patients starting PD. Time-dependent multivariate analysis.

	HR	95% CI	p
Age (x year)	1.06	1.04–1.07	<0.0005
Charlson’s score (x point)	1.07	0.98–1.18	0.18
Diabetes	1.80	1.21–2.66	0.003
Plasma albumin (x g/L)	0.94	0.91–0.97	<0.0005
Hemoglobin (x g/dL)	0.82	0.71–0.92	0.001
Peritoneal transport (x point)	1.01	1.00–1.03	0.07
Proteinuria (x g/24 hours)	1.09	0.04–0.15	0.001
GFR, time-dependent (x mL/min)	0.99	0.99–1.00	0.011
Event peritonitis, time dependent	1.00	1.00–1.00	0.74

Time-dependent Cox’s model. Outcome variable: mortality (any cause). Other time-dependent variables scrutinized not significant. GFR: Glomerular filtration rate (mean of urea and creatinine renal clearances).

### 3. PD technique failure

Our analysis confirmed the well known difficulties involved in the development of predictive models for PD technique failure. Univariate analysis did not show a significant effect of baseline GFR (p = 0,68 log rank test), and just a trend without significance (p = 0.08) for its rate of decline. General Cox’s model (Table 6) did show a modest effect of the pace of decline of RKF, which was the only identifiable, independent predictor of technique failure, with a minor trend for baseline GFR to have a similar effect (p = 0.22 for the interaction term). Time-dependent, multivariate analysis was consistent with these observations (Table 6).

**Table 6:** Predictors of PD technique failure. General and time-dependent multivariate analyses.

	Adjusted HR	95% CI	P
**General model**			
Baseline GFR (x mL/min)	0.92	0.84–1.01	0.083
Rate of decline of GFR (x mL/min/month)	1.49	1.01–2.20	0.043
**Time-dependent model**			
Age (x year)	0.98	0.964–0.99	0.015
GFR, time-dependent	0.99	0.991–1.00	0.060

Outcome variable: PD technique failure (drop-out to hemodialysis). GFR: Glomerular filtration rate (mean of urea and creatinine clearances).

### 4. Risk of peritoneal infection

Univariate analysis demonstrated a significant association between baseline GFR and the risk of peritoneal infection (p = 0.032, log rank test), with a similar trend (p = 0.068) for the rate of decline of RKF. General Cox’s model identified both baseline GFR (HR 0.94 per mL/minute, 95% CI 0.90–0.98, p = 0.008) and its rate of decline during follow-up (HR 2.08 per mL/minute/month, 95% CI 1.08–4.02, p = 0.028) as independent predictors of the risk of peritoneal infection (p = 0.75 for the interaction term), after controlling for other independent predictors of this complication (age, plasma albumin and C reactive protein). Time-dependent GFR was not a predictor of the risk of peritonitis (adjusted HR 1.06, 95% CI 0.99–1.15, p = 0.11). We did not detect interactions between GFR and age, serum levels of albumin, C reactive protein or modality of PD, as predictors of peritoneal infection.

## Discussion

The results of our study demonstrate that baseline GFR and its rate of decline during follow-up are independent predictors of outcome in patients starting PD therapy. As expected, baseline GFR and its rate of decline correlated, one positively and the other negatively, with mortality. A high baseline GFR portended a relatively fast decline in renal function, during follow-up (Fig 1). This type of correlation has been observed in some previous studies [13,16,17] and that can be explained, at least partly, as a mathematical consequence of expressing the decline of GFR in absolute terms. Consistent with this interpretation, a complete loss of RKF during follow-up is less likely if the baseline GFR is high [17–20].

More surprisingly, we detected a significant interaction between the effects of baseline GFR and its rate of decline on mortality. Considered in clinical terms, a higher baseline GFR offered a survival advantage only if the ensuing rate of GFR decline was indolent. A low baseline GFR, however, amplified the negative effect of a fast rate of GFR decline, during follow-up. Overall, stratified analysis of our data (Table 4) suggests that a fast decline of GFR is more consequential than baseline GFR as a predictor of mortality, because patients who started PD with a lower level of GFR, but experienced a slow rate of GFR decline during follow-up, did not have a greater risk of mortality. These findings confirm the independent effect of these study variables, but also the need to consider their interaction, for survival analyses.

Patient mortality was the outcome most clearly influenced by the study variables (Tables 3 to 5). This finding agrees with a majority of previous studies on this question [4–12]. Interaction analysis also revealed that the rate of decline of GFR had a more apparent influence on the risk of mortality in younger patients. This finding may be explained by the confounding effects of aging itself or its related comorbidities on survival.

Our data confirm the difficulties involved in modeling PD technique failure (Table 6), as reported by in previous studies [12,13]. Even when these difficulties are considered, baseline GFR and, particularly, its rate of decline performed well as consistent predictors of PD technique failure. This finding is not unexpected, because RKF mitigates the effects of the main complications resulting in PD drop-out [1]. For instance, RKF permits incremental dosing of PD, optimizing quality of life and reducing technique drop-out for social reasons. In addition, RKF helps to prevent dialysis inadequacy, volume overload, peritoneal membrane injury (lower peritoneal glucose load) and peritoneal infections. The results of our study indeed agree with previous reports suggesting an association between RKF and the risk of peritoneal infection [21]. This notwithstanding, we were unable to confirm this association using a time-dependent approach. The explanation for this discrepancy is not clear, although the relationship between RKF and peritoneal infection may be complex and bidirectional, because peritoneal infections may themselves accelerate the rate of decline of GFR (Table 5) [22–24]. Remarkably, aminoglycosides were not routinely used for the treatment of peritonitis in any of the participating centers.

The last decade has seen substantial controversy, regarding the advantages of an early *versus* late initiation of dialysis therapy. In general, observational studies of hemodialysis patients have shown an association between early start and increased mortality, during follow-up [14]. The interpretation for this unexpected finding is not totally clear. Hemodialysis therapy itself may carry negative effects for many patients, including an accelerated decay of RKF. The paradox, however, is commonly attributed to study selection biases, because early start of dialysis is more likely in high risk patients, where other comorbidities may conceal any positive effect of RKF on survival. Studies focusing exclusively on patients beginning PD therapy generally show a beneficial effect of doing so while the level of RKF is still high [14]. The reasons for the discrepancy between hemodialysis and PD are, once again, unclear. Differences in predialysis management could bear some influence, because the selection bias for early start may be less for PD than for hemodialysis patients. Additionally, incremental PD prescription may help prevent some adverse effects of hemodialysis therapy. In particular, a slower decline of RKF [1] could augment the potential benefits of starting dialysis at relatively high levels of GFR. A third explanation may lie with the different methods used to quantify GFR. In general, studies on PD patients tend to use the mean of urea and creatinine renal clearances, for this purpose. Studies on hemodialysis patients, on the other hand, calculate GFR from estimative, creatinine-based formulas, which tend to overestimate GFR in patients with a low muscle mass [25]. Notably, the randomized trial IDEAL was unable to demonstrate a prognostic significance for RKF at the initiation of dialysis [26], either in patients on hemodialysis or PD [15]. The results of this study, however, are also inconclusive, because of methodologic limitations, that include estimation of GFR according to the Cockroft-Gault formula and a high incidence of protocol violations.

The main limitation of our study resides with its retrospective, nonrandomized design. Statistical power was limited, particularly when it came to perform some subanalyses. Follow-up for GFR was closed at 24 months because, the number of subjects lost to follow-up limited the reliability of data. The main target of this study was to characterize simultaneously the prognostic significance of baseline and evolutionary GFR. Consequently, only patients with a significant RKF at the initiation of PD (arbitrarily set at ≥1 mL/minute). This peculiarity of the study design must be taken into account when comparing our results with those from other studies that included patients who were oligoanuric since inception of PD. Among its strengths, our study had a multicenter design, included thorough clinical information and provided well-defined results, that allowed us to address the question we set off to answer.

In summary, RKF at the initiation of PD and its rate of decline during follow-up are independent predictors of mortality, technique failure and peritoneal infection. These two variables maintain a moderate correlation, and interact clearly at the time of predicting patient survival. Our results indicate that the rate of decline of GFR has greater influence on mortality. In fact, a lower baseline RKF does not portend mortality as long as GFR decreases slowly. Overall, the survival benefit of early PD initiation cannot be evaluated without taking into consideration the rate of decline of RKF, a principle that may help explain the variable effects of early PD reported in the literature.
